# Supplementary material for: Patterns of Intron Gain and Loss in Fungi
Source: PLoS Biol. 2004 Nov 30;2(12):e422. doi: 10.1371/journal.pbio.0020422 (PMC532390; doi:10.1371/journal.pbio.0020422)
Supplement: Table S1 — Also available at http://genes.mit.edu/NielsenEtAl/. (4.3 MB ZIP). [file pbio.0020422.st001.zip › NielsenEtAl/html/335.html]

AN0906.1.NCU02011.1.MG03668.1.FG09454.1


```
 CLUSTAL W (1.82) Multiple Sequence Alignments - Introns Inserted


Sequence 1: MG03668.1	876 aa
Sequence 2: FG09454.1	877 aa
Sequence 3: NCU02011.1	876 aa
Sequence 4: AN0906.1	872 aa
Alignment Length: 882 aa
Number Identitical Residues: 484 aa
Alignment Score (without introns) 23314


MG03668.1 	-MATSDITTVLTNSLSAD1ANLRHAAEQQLTQAAETNF0SLYLATLVTELANENAPGHIR
NCU02011.1	MEGSSDINTVLTNSLSPD1ATLRHAAEQQLSQAAQTNF0SQYLVTLVQELANESAQSHIR
FG09454.1 	-MSSSEINQVLANSLSPD1ANLRNAAEQQLTQAAESNF0PLYLATLVQELANDSADGSIR
AN0906.1  	----MDVTQVLANTISSD1ANTRSNAEQQLLHAAEVDF0AGYLVTLGQELANENSASHIR
          	     ::. **:*::*.* *. *  ***** :**: :* . **.**  ****:.: . **

MG03668.1 	AAAGIAVKNAFTAREFSRQTELQQKWLQQTDDETRAKVKSLTLQTLSSTNSQAGQAAAQV
NCU02011.1	AAAGIALKNAFSAREFARQAELQAKWLQQTDQDTKTRVKQLTLETLASSSTQASQASAQV
FG09454.1 	AAAGIALKNAFTTRDFARHQELQAKWLQQTDDETKNRVKELTLQTLSSSNTQAGTAAAQV
AN0906.1  	TAAGLALKNAFTFRDRERLNEVQQKWRQQITPDIKAQVKELALKTLASKDGRAGQSAAQF
          	:***:*:****: *:  *  *:* ** **   : : :**.*:*:**:*.. :*. ::**.

MG03668.1 	IASIAGIELPRGQWADLMNILVTNVSEGQPHQKQASLTTIGFICESQDPELRASLVDHSN
NCU02011.1	IAAIATIELPRNEWPDLMHALVKNVSEGSEHQKQASLTTIGFICESQDVDLRNSLVQHSN
FG09454.1 	ISSIAAIELPRDQWNDLLPFLVKNVSEGADHQKQASLTTIGYICESQDSELRMALVTHSN
AN0906.1  	IVSIAAIELPQNEWPDLMQILVQNVASGSDQMKQASLVTIGFICESQEMELRESLAAHSN
          	* :** ****:.:* **:  ** **:.*  : *****.***:*****: :** :*. ***

MG03668.1 	AILTAVVQGARKEETNNEIRLAAITALGDSLEFVGNNFKHEGERNYIMQVVCEATQGDDS
NCU02011.1	AILTAVVQGARKEEPNREVRLAAITALGDSLEFVGNNFKHEGERNYIMQVICEATQAEDS
FG09454.1 	AILTAVVQGARKEETNIEVRLAAITALGDSLEFVGNNFKHEGERNYIMQVVCEATQADDS
AN0906.1  	AILTAVVQGARREEQNMDIRFAAIKALSDSVDFVRSNMENEGERNYIMQVVCEATQAEDL
          	***********:** * ::*:***.**.**::** .*:::**********:*****.:* 

MG03668.1 	RIQQGAFGCLNRIMALYYENMRFYMEKALFGLTILGMKSEDEDVAKLAVEFWSTVCEEEI
NCU02011.1	RIQQGAYGCLNRIMALYYENMRFYMEKALFGLTILGMKSDDEDVAKLAVEFWSTVCEEEI
FG09454.1 	RIQQGAFGCLNRIMALYYENMRFYMEKALFGLTILGMKSDDEDVAKLAVEFWSTVCEEEI
AN0906.1  	RVQAGAFGCLNRIMGAYYDKMSFYMEKALFGLSIMGMKSEEEDVAKLAIEFWCTVCEEEI
          	*:* **:*******. **::* **********:*:****::*******:***.*******

MG03668.1 	AIEDDN--~AQ~VESADQVRPFYNFARVATNEVVPVLLTLLTKQDEDAADDEYNISRAGY
NCU02011.1	AIEDDN--~AQ0VESSEQMRPFYNFARVATLEVVPVLLQLLTKQDEDAADDEYNISRAAY
FG09454.1 	SIEDDN--~AQ~VESSDQMRPFYNFARVAANEVVPVLLLLLTKQDEDATDDEYNLSRAAY
AN0906.1  	AIEDDNAA0AQ~AEGLTDVRPMYGFARIACREVVPVLLQAMCKQDEDAGDDEYNISRAAY
          	:*****:: ** .*.  ::**:*.***:*  *******  : ****** *****:***.*

MG03668.1 	QCLQLYAQAVGGTIIPPVISFVEGNLRSDDWHNRDAAVSAFGAIMEGPDEKTLEPIVKSA
NCU02011.1	QCLQLYSQAVGAAIIQPVIQFVEANLRADDWHLRDAAVSAFGAMMDGPEEKLLEPIVKSG
FG09454.1 	QCLQLYAQAVGATIISPVLSFVESNLRHEDWHNRDAAVSAFGAIMEGPDEKVLDPIVKQA
AN0906.1  	QALQLYAQCVQADVIQPVLAFVEENIRSEDWRRRDAAVAAFGAIMDGPDPKVLEPLVKQA
          	*.****:*.* . :* **: *** *:* :**: *****:****:*:**: * *:*:**..

MG03668.1 	LQILISMMDDSSVHVKDSTAYALGRITEACSEAIDPSQHLEPLIRSLFAGLLNTPKMAAS
NCU02011.1	MQPLIGMMEDPSLHVRDSTAYALGRITETCSEVIDPAVHLDPLITSLFNGLMSSPRMAAS
FG09454.1 	LPILITMMEDQSLHVKDSTAYALGRVTDACSEAIDPQAQLPTLIESLFKGLLSSAKMAPS
AN0906.1  	LHVLVSMMEDSSIQVRDSAAYALGRVCDFCSETLDPDVHLQPLISCLFNGLASSPKIASS
          	:  *: **:* *::*:**:******: : ***.:**  :* .** .** ** .:.::*.*

MG03668.1 	CCWALMNLAERFAGEPGAPQNAITAYFNDSVRSLLDVTAK~NDCDSAVRTAAYEVLNAFI
NCU02011.1	CCWALMNLAERFGGEYGAAQNPITPHFNQCVTNLLAVTAK~LDGDATVRTAAYEVLNVFV
FG09454.1 	CCWALMNLAERFAGDFGAASNAITPHFNNAVSSLLDVTAR~QDAETSVRTASYEVLNVFV
AN0906.1  	CCWALMNVADRFAGDVGAQTNPISKYFEESVKSLLALTER2SDADNQLRTAGYEVLNSFV
          	*******:*:**.*: **  *.*: :*::.* .** :* :  * :  :***.***** *:

MG03668.1 	VNAANDSLQAVATLSDVIIKRLEETIPLQTQVVSVEDRITLEDMQTSLSTVLQ~AIIGRL
NCU02011.1	QNAANDSLPAVASLSDVILQRLEETLPLQSQVVSVEDKITLEDMQTSLCTVLQ~AIIQRL
FG09454.1 	QNAASESLQPIASLSDVIIKRLEETVPLQSQVVSVEDKMTLEEMQNSLCSVLQ~AIISRL
AN0906.1  	TNAANDSLPTVAHLSDVVLQRLERTIPMQQQVVSVEDRIMLEEMQTGITSVVL0AIVQRL
          	 ***.:** .:* ****:::***.*:*:* *******:: **:**..: :*:  **: **

MG03668.1 	DKEILPQGDRIMQVLLQILSTVNGKSTVPEAIFATISSLANAIEEEFVKYMDAFAPFLYN
NCU02011.1	DKEITPQGDRIMQVLLQLLNTINGKSAVPEGVFAAISGLANAMEEDFAKYMDAFAPFLYN
FG09454.1 	DKEIIPQGDRIMQILLSILNSVGGKSSVPDAVFATISALSTAMEEDFVKYMDAFAPFLYN
AN0906.1  	EAEIKPQADRIMQILLQVLSTVPPKSSVPDVVFATVGAIANALEEEFVKYMESFSPFLNG
          	: ** **.*****:**.:*.::  **:**: :**::..::.*:**:*.***::*:*** .

MG03668.1 	ALGNQEEPSLCSMAIGLVSDITRSMGERSQPYCDNFMNYLLNNLR0--SSTLSNQFKPAI
NCU02011.1	ALANQEEPSLCSMAIGLVSDITRSMGERSQPYCDQFMNYLLNNLR~--STALANQFKPAI
FG09454.1 	ALGNQEEPSLCSMAIGLVSDITRSLGERSQPYCDNFMNYLLNNLR0SQSTALANQFKPAI
AN0906.1  	ALGNQEEPGLCAMAIGLVSDISRALNEKVLPYCDTFMNHLMNNLS0----SATNQLKPAI
          	**.*****.**:*********:*::.*:  **** ***:*:***      : :**:****

MG03668.1 	LQCFGDIANAIGGHFETYLSVVAQVLQQASTVTTVPEGSYEMFDYVVSLREGIMDAWGGI
NCU02011.1	LQCFGDIAGAIGGHFEAYLSVVAVVLQQAATVTASAEGSYEMFDYVISLREGIMDAWGGI
FG09454.1 	LQCFGDIAGAIGGHFETYLSVVAQVLEQASTVTASPEGPYEMFDYVVSLREGIMDAWGGI
AN0906.1  	LETFGDIAQAIGEHFDKYLTVVGQVLKQASLVTASNDVTIEMLDYIISLREGIMDAWGGI
          	*: ***** *** **: **:**. **:**: **:  : . **:**::*************

MG03668.1 	IGAMKSANKT1QALQPYVPSIFELLNHIGSD-SNRSESLMRSSMGVIG2DLADAYPNGEL
NCU02011.1	IGAMKGSDKT1NVLEPYVQSIFELLNTIAQD-PNRSEALMRAAMGVIG2DLADAYPNGQL
FG09454.1 	IGAMKVSQKT1QALQQYVPVIFNVLNLIAND-MNRSESLMRASMGVIG2DLADAYPNGEL
AN0906.1  	LLAYKGKPQA1QALKEFIDPIFELLRLISQDPASRSEGLMRASMGVLG~DLAETYPDGSI
          	: * *   :: :.*: ::  **::*. *..*. .***.***::***:* ***::**:*.:

MG03668.1 	VDAFRQDWVTAIIKETRSNREFSSRTIETARWAREQVKRQLGGSVNVMQQT
NCU02011.1	AEVFRQDWITAMIKETRSNREFQQRTIETARWAREQVKRQISGTQGMIQT-
FG09454.1 	VDAFRQDWVTAMIKETKTNREFQPRTIETARWAREQVKRQLGGSQAVMAN-
AN0906.1  	SAYFRNEWVTSLVRETRTNREYGQRTIDTARWAREQVKNQINMQGGGMS--
          	   **::*:*::::**::***:  ***:**********.*:.     :
```
